# Global Distribution of Extended Spectrum Cephalosporin and Carbapenem Resistance and Associated Resistance Markers in *Escherichia coli* of Swine Origin – A Systematic Review and Meta-Analysis

**DOI:** 10.3389/fmicb.2022.853810

**Published:** 2022-05-10

**Authors:** Shivdeep Singh Hayer, Alejandro Casanova-Higes, Eliana Paladino, Ehud Elnekave, Andre Nault, Timothy Johnson, Jeff Bender, Andres Perez, Julio Alvarez

**Affiliations:** ^1^Department of Veterinary Population Medicine, College of Veterinary Medicine, University of Minnesota–Twin Cities, St. Paul, MN, United States; ^2^Department of Biology, College of Arts and Sciences, University of Nebraska Omaha, Omaha, NE, United States; ^3^Departamento de Patología Animal, Facultad de Veterinaria, Universidad de Zaragoza, Zaragoza, Spain; ^4^Koret School of Veterinary Medicine, The Robert H. Smith Faculty of Agriculture, Food and Environment, The Hebrew University of Jerusalem, Jerusalem, Israel; ^5^Health Sciences Library, University of Minnesota–Twin Cities, Minneapolis, MN, United States; ^6^Department of Veterinary and Biomedical Sciences, College of Veterinary Medicine, University of Minnesota–Twin Cities, St. Paul, MN, United States; ^7^School of Public Health, University of Minnesota–Twin Cities, Minneapolis, MN, United States; ^8^VISAVET Health Surveillance Centre, Universidad Complutense Madrid, Madrid, Spain; ^9^Department of Animal Health, Facultad de Veterinaria, Universidad Complutense Madrid, Madrid, Spain

**Keywords:** antimicrobial resistance, pigs, cephalosporin, carbapenem, ESBL, *amp*C, *Escherichia coli*, systematic review

## Abstract

Third generation cephalosporins and carbapenems are considered critically important antimicrobials in human medicine. Food animals such as swine can act as reservoirs of antimicrobial resistance (AMR) genes/bacteria resistant to these antimicrobial classes, and potential dissemination of AMR genes or resistant bacteria from pigs to humans is an ongoing public health threat. The objectives of this systematic review and meta-analysis were to: (1) estimate global proportion and animal-level prevalence of swine *E. coli* phenotypically resistant to third generation cephalosporins (3GCs) and carbapenems at a country level; and (2) measure abundances and global distribution of the genetic mechanisms that confer resistance to these antimicrobial classes in these *E. coli* isolates. Articles from four databases (CAB Abstracts, PubMed/MEDLINE, PubAg, and Web of Science) were screened to extract relevant data. Overall, proportion of *E. coli* resistant to 3GCs was lower in Australia, Europe, and North America compared to Asian countries. Globally, <5% of all *E. coli* were carbapenem-resistant. Fecal carriage rates (animal-level prevalence) were consistently manifold higher as compared to pooled proportion of resistance in *E. coli* isolates. *bla*_*CTX–M*_ were the most common 3GC resistance genes globally, with the exception of North America where *bla*_*CMY*_ were the predominant 3GC resistance genes. There was not a single dominant *bla*_*CTX–M*_ gene subtype globally and several *bla*_*CTX–M*_ subtypes were dominant depending on the continent. A wide variety of carbapenem-resistance genes (*bla*_NDM–, VIM–, IMP–, OXA–48_, _and_ _KPC–_) were identified to be circulating in pig populations globally, albeit at very-low frequencies. However, great statistical heterogeneity and a critical lack of metadata hinders the true estimation of prevalence of phenotypic and genotypic resistance to these antimicrobials. Comparatively frequent occurrence of 3GC resistance and emergence of carbapenem resistance in certain countries underline the urgent need for improved AMR surveillance in swine production systems in these countries.

## Introduction

Third generation cephalosporins (3GCs) such as ceftriaxone and cefotaxime are used to treat Gram-negative infections in humans (Iwamoto et al., 2017) and are considered critically important antimicrobials in human medicine according to the World Health Organization., 2019. Consequently, resistance to these antimicrobials has received increased attention in molecular epidemiological studies in bacteria retrieved from humans (Manges et al., 2019). Third generation cephalosporins are also used in food animal production including swine for treating respiratory disease complex (Hayer et al., 2020b; Sun et al., 2020). Although some of these antimicrobials such as ceftiofur are only used in food animal medicine, their use can exert selective pressure favoring resistance to cephalosporins used in human medicine (Cantón and Ruiz-Garbajosa, 2011). Hence, there is a need to monitor the prevalence of resistance to higher generation cephalosporins in swine production in order to monitor the role of swine production systems as a reservoir of potentially zoonotic bacteria resistant to these antimicrobials. Resistance to 3GCs is mediated by extended spectrum beta-lactamases (ESBLs, commonly coded by *bla*_*SHV–* and CTX–M–_ family of genes) and *amp*C (commonly coded by *bla*_*CMY*_ family of genes) (Pfeifer et al., 2010). These genes can be plasmid-borne and plasmids aid in the widespread dissemination of these genes horizontally or across different bacterial species as well as mammalian hosts (Hayer et al., 2020a).

The global spread of pathogens resistant to third and fourth generation cephalosporins has led to an increased consumption of last resort antimicrobials in human medicine such as carbapenems (Woodford et al., 2014). Carbapenemases are β-lactamase enzymes capable of hydrolyzing not only carbapenems but also penicillins and 3GCs, and genes encoding these enzymes (like *bla*_VIM–, IMP–, OXA–48, NDM–,_ _and_ _GES–_) can also be transmitted between different bacterial species *via* plasmids (Giske et al., 2009; Nordmann et al., 2012). Although carbapenems are not licensed for use in food animal production in any country (Woodford et al., 2014), the use of licensed higher generation cephalosporins such as ceftiofur in animals can lead to co-selection of carbapenem resistance (Mollenkopf et al., 2017).

In order to make a reliable assessment of the possible role of antimicrobial usage in animals on the emergence of antimicrobial resistance (AMR) and its impact on human health, knowledge on the occurrence of AMR in different animal production systems is needed. The aim of this systematic review was (1) to evaluate available information on the frequency and types of phenotypic resistance to 3GCs and carbapenems in swine *Escherichia coli*, and (2) to assess the spread of select *bla* genes conferring these resistance phenotypes in this bacterial species. *E. coli* was selected as an indicator bacterial species because of its widespread prevalence across multiple animal species, ease of isolation and propensity to develop resistance against antimicrobials reviewed in this study (Tadesse et al., 2012) and has been identified as the most relevant resistant bacterial pathogen in swine populations (Nielsen et al., 2021).

## Methods

### Literature Search

Four databases, PubAg, Web of Science, PubMed, and CAB Abstracts, were screened using different search strings (Supplementary File 1) on 13 April 2017 and were updated on 18 October 2021. Search strings were designed to identify publications on cephalosporin, colistin, quinolone, and carbapenem resistance in swine. Data on fluoroquinolone and colistin resistance was extracted to conduct a separate systematic review (Hayer et al., 2022).

Records were retrieved, and duplicate articles were removed using Zotero version 5.0.96.3. In addition to the database searches, reports containing information on AMR in *E. coli* from swine were retrieved from the websites of national and international agencies coordinating surveillance programs for AMR (EFSA, Europe; RESAPATH, France; DANMAP, Denmark; MARAN, Netherlands; NORMVET, Norway; SWEDRES, Sweden; FINRES, Finland; NARMS, United States; and CIPARS, Canada).

Retrieved records were first screened for inclusion if there was a mention of AMR, *E. coli* or *Enterobacteriaceae*, and livestock in title and/or abstract. The selected full-length articles were then downloaded and data was collected from articles if there was data on occurrence of phenotypic AMR against 3GCs or carbapenems in *E. coli* collected from pigs. Articles were excluded if:

• The data was based on *in vivo* or *in vitro* trials that carried out experiments other than measuring phenotypic or genotypic AMR in *E. coli* circulating in swine populations. For example, if an animal trial involved feeding antimicrobials to pigs and then studying occurrence of AMR, then that study was excluded. Similarly, studies that included experimental induction of AMR in *E. coli* strains *in vitro* were excluded.
• Articles were written in a language other than English
• Articles were providing a review, a commentary or data on just antimicrobial usage
• Data was not provided for *E. coli* specifically
• Bacteria were not isolated from pigs
• Data was based on a single farm/outbreak, with the exception of articles that presented research based on multiple farms but were able to find an isolate of interest from a single farm
• Article described a single isolate that carried a gene of our interest but did not provide the number of isolates tested (lack of denominator)
• A metagenomic approach was taken
• Total number of isolates tested was not provided.

### Data Extraction

A predesigned datasheet was developed using Microsoft Excel 2013 for collecting data such as publication year, country and year of bacterial isolation, health status of pigs, phenotypic resistance tested, microbiological methods employed for AMR determination, criteria for classifying bacteria as resistant/non-resistant, total number of isolates tested and number of isolates resistant against each antimicrobial and whether information on sampling design, antimicrobial use and husbandry was provided. Extracted phenotypic and genotypic data are provided in Supplementary File 2.

### Meta-Analyses of Literature Review

Arguably, the biggest issue in aggregating AMR data across studies was the use of different breakpoints to classify bacteria as “resistant” in these studies. We harmonized the AMR prevalence data in a multi-step manner. First, for those studies where MIC distributions were available, the number of resistant bacteria were re-calculated as if the current CLSI breakpoints (CLSI, 2021) were used for defining resistance. If MIC distributions were not available or disk diffusion method was used for estimating resistance, the number of resistant isolates in a particular study was multiplied by a harmonizing factor. These harmonization factors were calculated using the following formulae (Van Boeckel et al., 2019):

1. For dilution methods – number of isolates in the reference EUCAST distribution with MIC ≥ current CLSI breakpoint divided by number of isolates in reference EUCAST distribution with MIC ≥ applied breakpoint in the study from which data were extracted.
2. For diffusion methods – number of isolates in reference EUCAST distribution with zone of inhibition ≤ current CLSI breakpoint divided by number of isolates in reference EUCAST distribution with zone of inhibition ≤ applied breakpoint in the study from which data were extracted.

MIC or disc diffusion reference distributions were downloaded from EUCAST (2021). Breakpoints were determined for each article based on the performance methodology (e.g., CLSI-M100 series) document provided in the bibliography. If this information was not provided, it was assumed that authors used CLSI breakpoints for interpretation as these are the most commonly used guidelines to differentiate resistant bacteria globally (Van Boeckel et al., 2019).

Considering that isolates collected from healthy pigs were likely to have different phenotypic resistance profiles compared to isolates from diseased pigs, pooled prevalences of resistance were estimated separately for these health categories at a country level. Studies that reported AMR levels in mixed bacterial populations isolated from both healthy and diseased pigs were not included in the meta-analyses, but the results from these studies are available in Supplementary File 2. Studies that did not provide data on health status were assumed to have been conducted on healthy pigs because the authors compared their results to those from other healthy pig populations in the discussion section and these isolates were not based on diagnostic submissions or collected during outbreak investigations. Removing data from such articles did not impact the pooled prevalence estimates (data not shown).

Another issue with pooling resistance data was the detection of resistance to different 3GCs and carbapenems across studies. To address this issue, we selected three 3GC resistances as markers: ceftiofur, cefotaxime, and ceftriaxone resistance. At least one of these cephalosporin resistances was estimated in all studies except one, and for studies estimating resistance to more than one of these 3GCs, the correlation between the proportions of isolates resistant to these 3GCs was high (correlation coefficients > 0.9). For those studies providing data on more than one of these three cephalosporin resistances (e.g., both ceftiofur and cefotaxime resistance measured on same isolates), we used the highest number of isolates estimated to be resistant after harmonization for any of these 3GCs in a study for downstream analyses. For example, if the number of ceftiofur and cefotaxime resistant isolates after harmonization were 30 and 35, respectively in a study, then we used 35 as the number of isolates resistant to 3GCs for statistical analysis. In addition, when available, ceftazidime resistance was analyzed separately from the other 3GCs because the genetic basis of ceftazidime resistance and ceftiofur, cefotaxime, or ceftriaxone resistance are different (Hiki et al., 2017). Similarly, if a study provided data on multiple carbapenem resistances (e.g., doripenem and ertapenem resistance for same set of isolates in the same article), the highest number of isolates after harmonization for any of these carbapenems were used as a proxy for number of isolates resistant to carbapenem on a per article basis.

The pooled proportions of 3GC, ceftazidime and carbapenem resistances were calculated at a country-level by transforming the proportions of resistant isolates using the Freeman–Tukey double arcsine transformation method (Miller, 1978), followed by a random effects model using inverse method and back-transforming to get the final pooled proportion estimates (in percent) across studies using *library meta* version 5.1-0 (Schwarzer, 2021). Statistical heterogeneity (*I*^2^) was retrieved from the output of this model and pooled estimates at a country-level were considered to be heterogenous and homogenous if *p*-values were <0.05 and >0.05, respectively. Choropleth maps were created to graph the pooled proportions of 3GC and ceftazidime resistances at a global scale using QGis version 3.20.3.

Similarly, pooled fecal carriage rates of 3GC and carbapenem resistance were also estimated and mapped. Fecal carriage rate is defined as the number of fecal samples collected from individual animals that test positive for carrying *E. coli* with specific resistance phenotype after screening on selective media. Herd level prevalence was not estimated and are not presented in the results section but raw data to do so has been provided in Supplementary File 2. Finally, choropleth maps for estimates for “sampling index” were also generated. For this study, sampling index was defined as the number of unique isolates tested for phenotypic 3GC, ceftazidime or carbapenem resistance divided by the number of specific pig population at a country level. For ease of interpretation, this index was generated per 1,000 pigs. Country-level pig population data (as of 2018) was downloaded from FAO (Food and Agriculture Organisation, 2022).

### Genomic Meta-Analyses

Data on genotypic prevalence of 3GC and carbapenem resistance was also extracted from the articles selected above and is available in Supplementary File 2. Pooled proportions of ESBL/*amp*C genes in *E. coli* isolated on selective media or having unique resistance phenotypes (i.e., carbapenem resistant, ESBL/*ampC* producers, and 3GC resistant) were estimated as described above.

In order to extract more data on the genomic basis of resistance to these antimicrobials, we downloaded information on genomes of 6,167 *E. coli* isolates of swine origin from Enterobase webserver (Zhou et al., 2020) (last accessed on 24 July 2021) and analyzed these genomes. The advantage of this data over the PCR data extracted from the literature search is that information regarding several genomic characteristics can be obtained at the isolate level. However, the disadvantage of this dataset is a lack of critical metadata such as microbiological methods employed, health status of the animal, etc.

Short read sequences of these isolates were downloaded from NCBI database based on their accession numbers from Enterobase (Zhou et al., 2020). Raw reads were checked for quality control using FastQC version 0.11.9 (Andrews, 2021) and subsequently trimmed using Trimmomatic version 0.39 (settings: sliding window mode; number of bases to average across = 4; average quality required = 20) (Bolger et al., 2014). Trimmed reads were then assembled using Shovill version 1.1.0 (default settings) (Seemann, 2021b). When short reads were not available for download, assembled genomes were directly downloaded from the Enterobase webserver. Genomes were characterized for virulence genes (Virulence Finder) (Malberg Tetzschner et al., 2020), resistance genes (ResFinder) (Bortolaia et al., 2020), chromosomal mutations that can confer resistance (PointFinder) (Zankari et al., 2017) and plasmid replicons (Plasmidfinder) (Carattoli et al., 2014) using Abricate version 1.0.0 (Seemann, 2021a). All databases were last updated on 24 July 2021. Isolates carrying ESBL/*amp*C and carbapenemase genes were identified and described in the sections below. However, the number of the isolates carrying carbapenemase resistance genes were too few to do the analyses described in the following paragraphs.

Relative abundance of ESBL/*amp*C genes were estimated as the ratio of isolates carrying a particular *bla* gene family (e.g., *bla*_*CMY*_) divided by total number of isolates carrying *bla*_*CMY*,_ *bla*_*CTX–M*_ and/or *bla*_*SHV*_. Furthermore, relative abundance of subclasses of *bla*_*CTX–M*_ genes were estimated as the ratio of isolates carrying a particular *bla*_*CTX–M*_ subclass gene family (e.g., *bla*_*CTX–M–*15_) divided by total number of isolates carrying *bla*_*CTX–M*_ genes. Relative abundances of ESBL/*amp*C genes (*bla*_*CMY*,_ *bla*_*CTX–M*_, and *bla*_*SHV*_) and *bla*_*CTX–M*_ subclasses at a global and continent level were plotted using *library ggplot2* version 3.3.5 (Wickham et al., 2021).

Random Forest models were built to characterize genetic features (virulence factors, plasmid replicons, chromosomal mutations, acquired resistance genes, and plasmid replicon types) that could predict the presence of *bla*_*CMY*_ and *bla*_*CTX–M*_ genes. Two models were built separately for *bla*_*CMY*_ and *bla*_*CTX–M*_ genes (dependent variables). Models were not built for *bla*_*SHV*_ isolates as they were too few in numbers to make reasonable models that can be generalized at a local or global scale. Only one of the perfectly correlated genetic features (*r* > 0.99) was retained for further analysis using *library caret* version 6.0-90 (Kuhn et al., 2021). The dataset was divided into training (65% of the dataset) and testing (35% of the dataset) data based on stratified random sampling in order to retain the same proportion of results for the dependent variables in these split datasets (presence of *bla*_*CMY*_ or *bla*_*CTX–M*_ depending on the model) (*library caTools* version 1.18.2) (Tuszynski, 2021). These models were first built for imbalanced training datasets using quantile-classifier algorithm (O’Brien and Ishwaran, 2019) in *library RandomForestSRC* (version 2.14.0) with the following settings: *mtry* = 25, number of trees = 12,000, split rule = AUC, block size = 100. Model performances were evaluated using geometric means. Training models were used to identify the top variables (i.e., variables of importance) that can predict *bla*_*CMY*_ or *bla*_*CTX–M*_ genes in testing data using default settings (*library RandomForestSRC* version 2.14.0) (Ishwaran and Kogalur, 2021). The top 10 variables of importance were extracted and plotted using *library ggplot2* version 3.3.5 (Wickham et al., 2021). Unadjusted odds ratios were also estimated for the top 10 variables of importance to estimate an effect size and directionality and for ease of interpretation.

## Results

### Description of Articles Included

**FIGURE 1:**
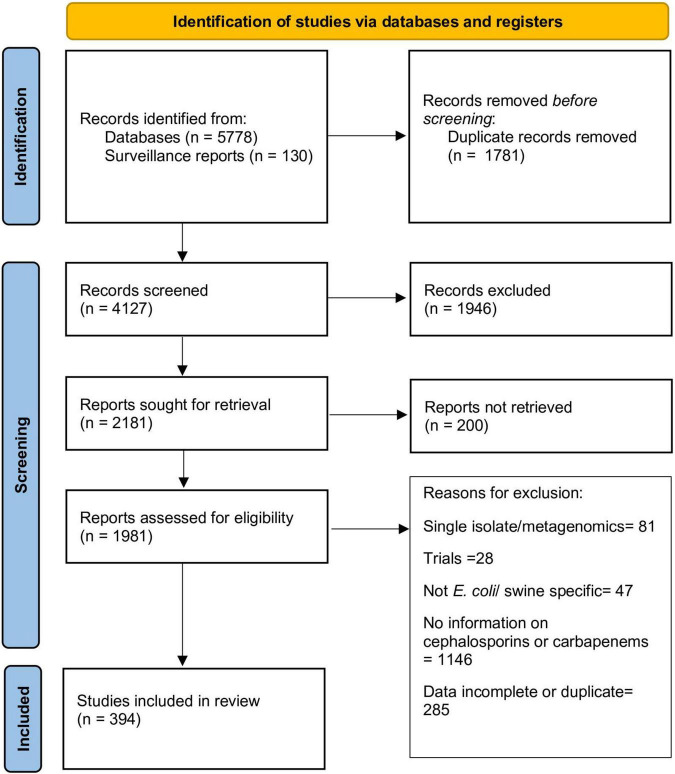
Flowchart describing selection of articles included in this review.

The stepwise process followed for article selection is presented in Figure 1. Ultimately, data from 394 articles that fulfilled the inclusion criteria was extracted and evaluated in this review. Description of these articles is presented in Table 1. The majority of the articles contained data from European (196 articles; 49.7%) or Asian (125 articles; 31.7%) countries. The studies included were published from 1998 to 2021, although there was a clear increase in the number of articles published after 2010 (70.1% of all articles). EUCAST epidemiological cut-offs (*n* = 83) and CLSI breakpoints (*n* = 174) were used to classify isolates as susceptible/resistant in 222 articles. The majority of the articles utilizing EUCAST epidemiological cut-offs were from European countries, whereas CLSI breakpoints were predominantly used in studies from other countries. Overall, data on resistance against 3GCs or carbapenem in *E. coli* of swine origin from 61 countries were available.

**TABLE 1:** General characteristics of the data reported in the articles selected reporting antimicrobial resistance data for cephalosporins and carbapenems in swine *E. coli* isolates (*n* = 394 articles).

Characteristic of articles	Specific feature	Number of articles
Phenotypic methods used for antimicrobial susceptibility typing	Diffusion	121
	Dilution	182
	Diffusion and dilution	2
	Unknown	2
Publication years	Before 2000	2
	2000–2005	49
	2006–2010	67
	2011–2015	109
	2016–2021	167
Time of sample collection	Known	354
	Unknown	40
Age of pigs	Described	293
	Unknown	101
Sample scheme	Described	207
	Not described	187
Management/husbandry	Described	67
	Not described	327
Antibiotic use	Mentioned	154
	Not mentioned	240
Breakpoint used for interpretation of resistance results	CLSI	174
	EUCAST	83
	Others	44
	Unknown	10
Geographic distribution of the articles	Africa	7
	Asia	125
	Europe	196
	North America	45
	Oceania	7
	South America	14
Geographic distribution of the number of isolates studied for phenotypic resistance	Africa	782
	Asia	31,088
	Europe	90,597
	North America	50,314
	Oceania	1,207
	South America	1,202
Specific location/geographic extent	Described	346
	Not described	48
Health status	Healthy	231
	Diseased	151
	Mixed	13
	Unknown	45

Information on health characteristics of the pigs, time of sample collection and specific geographic location was available in 88.8, 89.8, and 87.8% of the articles, respectively. However, information regarding the age of pigs, sampling scheme employed for isolate collection, husbandry characteristics and previous antibiotic use was only available in 74.4, 52.5, 17.0, and 39.1% of the articles, respectively. These figures on reporting characteristics further decrease if surveillance reports are not considered. For example, if we only include data published in journal articles, then only 19.4% of the studies reported previous antibiotic use. Although we did not analyze this formally, there were very few studies that provided data regarding the clinical history, tissues collected and strain information of the *E. coli* collected from diseased pigs.

**FIGURE 2:**
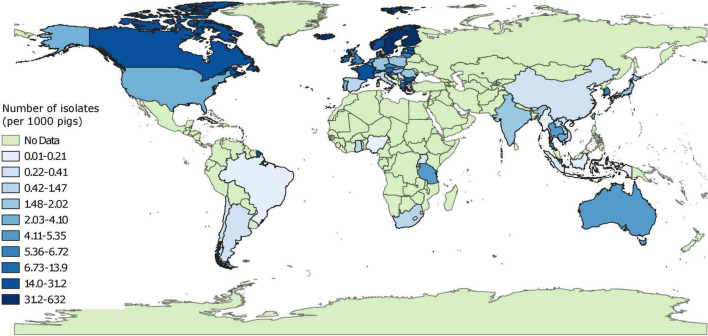
Global distribution of sampling indices (number of isolates characterized for phenotypic resistance per 1,000 pigs). “No data” implies that there were no isolates of interest reported and does not mean that there were no pigs in particular countries.

There was a clear disparity between number of isolates characterized phenotypically for 3GC, ceftazidime or carbapenem resistance across continents (Table 1). Overall, there were 175,190 isolates evaluated for phenotypic resistance globally, of which 51.7 and 28.7% of the isolates were collected from Europe and North America, respectively. After factoring in the pig populations by calculating sampling indices, this disparity was even more evident (Figure 2 and Supplementary File 2). Out of 15 countries with the highest sampling indices, 13 were European and rest of the countries were Canada and Grenada. On the other hand, 13 out of 15 countries with lowest sampling indices were Asian (6), African (4), or South American (3) and the rest of the countries were Cuba and Serbia. The exact number of isolates per study are provide in Supplementary File 2.

### Pooled Proportion Estimates of Third Generation Cephalosporin Resistance in Isolates From Healthy Pigs

**FIGURE 3:**
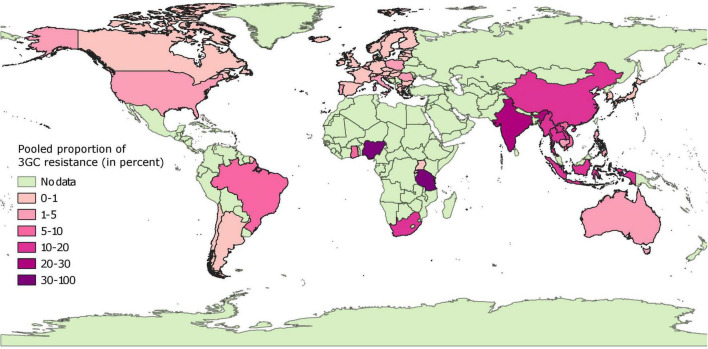
Global distribution of pooled proportion estimates of third generation cephalosporin resistance in *E. coli* isolates collected from healthy pigs. “No data” implies that there were no isolates of interest reported and does not mean that there were no pigs in particular countries.

Pooled proportion estimates of 3GC resistance in *E. coli* from healthy pigs at the country level are mapped in Figure 3 and provided in Supplementary File 3. Briefly, 3GC resistance was very low (0–1%) and low (1–2%) in isolates collected from 24 and 8 European countries, respectively, with isolates from Portugal having highest proportion of this resistance (2%). These estimates were statistically homogenous for data from 26 out of the 32 European countries. Similarly, the pooled proportion of 3GC resistance ranged between very low and low in countries from North America (Grenada, 0%; Canada, 1%; and United States, 3%) and Oceania (Australia, 2%). In South America, the pooled proportion estimates were also very low to low (Chile, 0%; Argentina, 0%; and Brazil, 8%). However, the estimates from North American, South American, and Oceanic countries were either based on one data-point or were statistically heterogenous, with the exception of pooled proportion of 3GC resistance in Argentina, which was statistically homogenous.

In contrast to the results above, the pooled proportion estimates from African and Asian countries were highly variable. Very low (<1%) pooled proportion estimates of 3GC resistance were estimated for Japan, South Korea, Rwanda, and Uganda. Low percentage of isolates were resistant to 3GCs in four countries (Cambodia, 2%; Philippines, 5%; Vietnam, 8%; and Ghana, 7%). In contrast, for other countries estimates of pooled proportion of 3GC resistance ranged between moderate (Laos, 12%; Indonesia, 12%; China, 16%; Myanmar, 18%; Thailand, 20%; and South Africa, 19%), high (India, 24% and Tanzania, 37%), and extremely high (Nigeria, 91%). These estimates were either statistically heterogeneous or based on a single data-point, except for Japan, Laos, and Uganda.

### Pooled Proportion Estimates of Third Generation Cephalosporin Resistance in Isolates From Diseased Pigs

**FIGURE 4:**
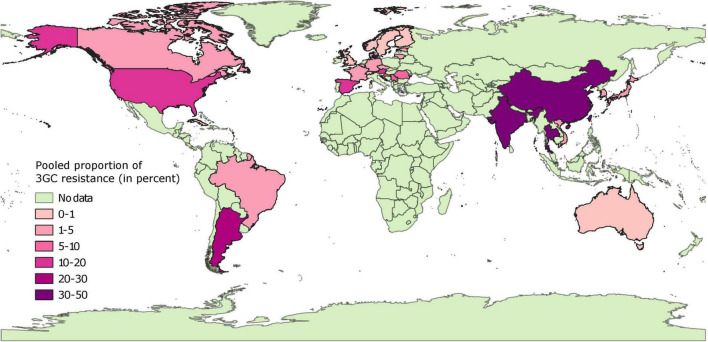
Global distribution of pooled proportion estimates of third generation cephalosporin resistance in *E. coli* isolates collected from diseased pigs. “No data” implies that there were no isolates of interest reported and does not mean that there were no pigs in particular countries.

Data on isolates from diseased pigs was available from 30 countries. Pooled proportion estimates of 3GC resistances at the country level are mapped in Figure 4 and provided in Supplementary File 3. In general, the pooled proportion estimates of 3GC resistance were higher in isolates from diseased pigs compared to healthy pigs. In Europe, North America, and Australia, pooled proportion in these subsets of isolates ranged between very low (<1% in 8 countries) and low (1–6% in 10 countries), with higher (moderate) pooled proportion estimated from Spain (14%), Austria (14%), and United States (19%). In Asian and South American countries, pooled proportion ranged from low (between 1 and 4% in South Korea, Brazil, Vietnam, and Japan) to high (between 24 and 47% in Argentina, India, China, Thailand, and Taiwan). No estimates from diseased pigs in African countries were available. The estimates were statistically homogenous for 10 of the 30 countries (8 European countries, Australia, and Taiwan).

### Pooled Proportion Estimates of Ceftazidime Resistance

Detailed pooled proportion estimates of ceftazidime resistance are presented in Supplementary File 3. Estimates of resistance to ceftazidime largely mirrored the estimates for frequency of 3GC resistances. Based on the data from 46 countries, the correlation between proportions of ceftazidime and 3GC resistance in isolates from healthy pigs were strong (*r* = 0.98, Pearson correlation). However, the pooled proportion estimates of 3GC resistance were 6.54 (95% CI: 2.57–10.5) times higher as compared to ceftazidime resistance in isolates collected from healthy animals. There was a moderate correlation between proportion estimates of 3GC and ceftazidime resistance in isolates from diseased pigs (*r* = 0.58, 10 countries, Pearson correlation), and proportion estimates of 3GC resistance were 1.93 times higher (95% CI: 0.07–3.79) than those of ceftazidime.

### Pooled Proportion Estimates of Carbapenem Resistance

Data on carbapenem resistance in isolates from healthy and diseased pigs was available from 47 countries (32 European, 7 Asian, 5 African, and 3 North American) and 9 countries (2 European, 6 Asian, 1 Australia), respectively. Proportions of carbapenem resistance in isolates from healthy pigs were estimated to be zero in 38 of these countries (Table 2). Pooled proportion of carbapenem resistance was 0% in isolates from healthy pigs from the European countries, except Portugal (0.1%). In Asia, these proportions ranged from 0% in Cambodia, Japan, and Laos, to 0.8–3.6% in China, Philippines, Thailand, and Vietnam. Estimates were highly variable in African countries ranging from 0% (Uganda and Rwanda), low (2% South Africa and 5 Tanzania) to 100% [Ghana, on a study performed on 43 isolates (Larbi et al., 2021)].

**TABLE 2:** Pooled proportion estimates of carbapenem resistance.

Health status	Country	Pooled proportion (95% CI)	Heterogeneity (*I*^2^ in percentage)
Healthy	China	0.88 (0–4.22)	94.60*
	Ghana	100 (96.0–100)	NA
	Philippines	3.64 (0.06–10.7)	NA
	Portugal	0.13 (0–0.61)	0
	South Africa	1.78 (0.22–4.45)	NA
	Tanzania	4.95 (2.44–8.25)	NA
	Thailand	1.37 (0–5.55)	97.1*
	Vietnam	2.6 (0.78–5.30)	42.8
	Countries with pooled proportions equal to zero	Rwanda, Uganda, Cambodia, Japan, Laos, Austria, Belgium, Bulgaria, Croatia, Cyprus, Czechia, Denmark, Estonia, Finland, France, Germany, Greece, Grenada, Hungary, Iceland, Ireland, Italy, Latvia, Lithuania, Luxembourg, Malta, Netherlands, North Macedonia, Norway, Poland, Romania, Slovakia, Slovenia, Spain, Sweden, Switzerland, United Kingdom, Canada and United States of America	
Diseased	China	0.62 (0–3.04)	56.3
	Europe	0.14 (0.07–0.24)	NA
	India	4.62 (0–23.8)	92.0*
	South Korea	1.09 (0–5.06)	31
	Thailand	59.1 (51.7–66.3)	NA
	Countries with pooled proportions equal to zero	Australia: Austria and Switzerland: Japan and Taiwan	

*Heterogeneity was statistically significant (p < 0.05).

*NA, estimates based on a single study and heterogeneity was not calculated.*

Pooled proportion estimates of carbapenem resistance were also 0% based on data from isolates from diseased pigs in Australia, Austria, Japan, Switzerland, and Taiwan (Table 2). In addition, a multi-country study in Europe estimated this proportion at 0.1% in isolates from diseased pigs in six countries (Germany, Belgium, United Kingdom, Denmark, Italy, and Netherlands) (Pulss et al., 2017). In this study, resistant isolates were isolated only from Germany and Italy. In China, India, and South Korea, this prevalence ranged between 0.6 and 4.62%, whereas in Thailand this prevalence was 59.1% based on a single study (Pokhrel et al., 2018; Table 2).

### Pooled Fecal Carriage Rates (Animal-Level Prevalence) of Third Generation Cephalosporin Resistance

**FIGURE 5:**
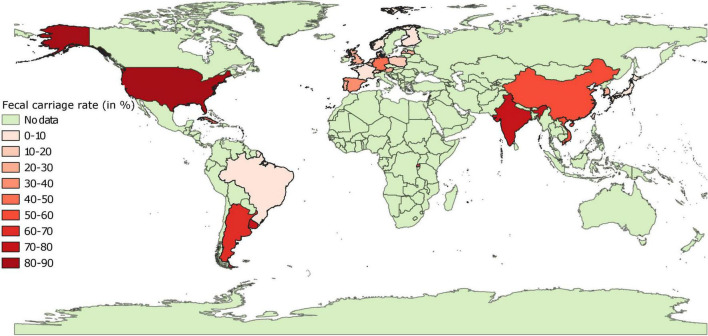
Global distribution of fecal carriage rates (animal-level prevalence) of third generation cephalosporin resistant *E. coli* isolates in pigs. “No data” implies that there were no isolates of interest reported and does not mean that there were no pigs in particular countries.

Data on pooled fecal carriage rates of 3GC resistance was available from 26 countries (Figure 5 and Supplementary File 3). Fecal carriage rates were lower in European countries as compared to most of the non-European countries. In general, fecal carriage rates were 433 times higher than the pooled proportion estimates. In Europe, pooled fecal carriage rates ranged were <10% in Finland, France, and Norway, between 10 and 20% in Switzerland, Denmark, Belgium, Poland, and Netherlands and between 20 and 45% in Latvia, United Kingdom, Portugal, Austria, Spain, and Germany. In Asia, pooled fecal carriage rates ranged between 5 and 22% in Japan, South Korea, and Taiwan, whereas this rate ranged between 55 and 80% in samples from Vietnam, China, Lebanon, and India. These rates ranged between 44 and 80% in other countries (Cuba, Rwanda, Argentina, Uruguay, and United States), with the exception of Brazil (3%). It should be noted that data from 17 of these 26 countries was based on a single study, and pooled estimates were statistically heterogenous for all but 3 countries (Finland, Japan, and Poland). Still, there was a moderate correlation (*r* = 0.53, Pearson correlation) between pooled proportion estimates and fecal carriage rates of 3GC resistance. Fecal carriage rate of carbapenem resistance was found to be zero in 6,791 fecal samples collected from Norway, Sweden, Denmark, Finland, and Switzerland. Pooled fecal carriage rate of carbapenem resistance was estimated to be 1.36% from China and India.

The biggest confounding factor in estimation of accurate fecal carriage rates was the use of different antibiotics and concentrations thereof in the media used for isolating 3GC or carbapenem *E. coli*. Of the 55 articles reporting animal-level or herd-level prevalence of 3GC resistance, 16 employed plates that were commercially available for 3GC detection and rest (39) added various 3GCs to routinely used lab media for 3GC detection (Supplementary File 2). Out of these 39 studies, 32 and 5 used cefotaxime and ceftriaxone at the concentration of 1–4 mg/L to supplement media, respectively. Rest of the studies used cefoxitin and ceftiofur (Supplementary File 2). Similarly, of the 14 articles that provided estimates of carbapenem resistance at herd- or animal-level, 9 used commercial plates and 5 supplemented routine laboratory media with ertapenem, meropenem and imipenem (Supplementary File 2).

### Relative Abundance and Genomic Characteristics of Extended Spectrum Beta-Lactamase*/amp*C and Carbapenemase Genes Based on Literature Search

Most of the studies focused on identifying genes of interest after selecting isolates on a media containing carbapenems or 3GCs or after identifying individual isolates as ESBL/*amp*C-producers. We have provided the data on isolates collected without aforementioned selection procedures in the Supplementary File 2, but we will only discuss data on those isolates that were identified based on selection procedures in the rest of this section.

**FIGURE 6:**
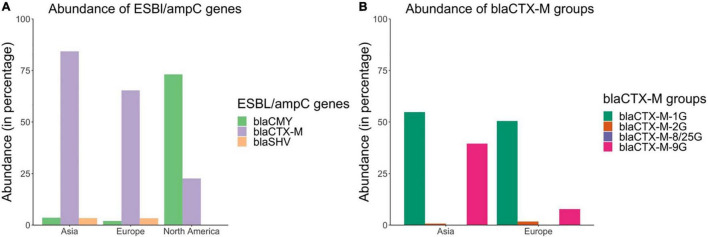
Abundances of **(A)** *bla*_*CTX–M*,_ *bla*_*CMY*_, and *bla*_*SHV*_ genes and **(B)** *bla*_*CTX–M*_ groups in ESBL/*amp*C or 3rd generation cephalosporin resistant *E. coli* isolates based on the literature review.

*bla*_*CTX–M*_ were the dominant genes in ESBL/*amp*C or 3GC-resistant *E. coli* isolates in Asia and Europe and were present in 84.3% (95% CI: 76.3–91.1%) and 65.3% (95% CI: 55.1–74.9%) of such isolates in Asia and Europe, respectively (Figure 6A). *bla*_*SHV*_ genes were present in 3.42% (95% CI: 0.08–9.55%) and 3.36% (95% CI: 1.09–6.44%) of ESBL/*amp*C or 3GC-resistant *E. coli* in Asia and Europe, respectively. *bla*_*CMY*_ genes were present in only 3.58% (95% CI: 0.19–9.41%) and 1.99% (95% CI: 0.55–3.99%) of such isolates in Asia and Europe, respectively (Figure 6A). In contrast, *bla*_*CMY*_ was the most prevalent gene group in 3GC-resistant *E. coli* in North America and these genes were present in 73.1% (95% CI: 41.9–95.4%) of the isolates; whereas *bla*_*CTX–M*_ and *bla*_*SHV*_ were present in 22.6% (95% CI: 0–65.7%) and 0% (95% CI: 0–1.41%) of such isolates, respectively (Figure 6A).

There were also differences in the distribution of *bla*_*CTX–M*_ groups across different continents (Figure 6B). For example, in Europe *bla*_*CTX–M–*1*G*_ (consisting primarily of *bla*_*CTX–M–*1, –15_, _–55_) was the predominant *bla*_*CTX–M*_ group and was present in 50.5% (95% CI: 40.2–60.7%) of the ESBL/*amp*C or 3GC-resistant *E. coli* isolates; whereas in Asia, both *bla*_*CTX–M–*1*G*_ (54.8%, 95% CI: 44.3–65.2%) and *bla*_*CTX–M–*9*G*_ (consisting of *bla*_*CTX–M–*14, –27_, _and__–65_) (39.5%, 95% CI: 29.7–49.5%) were the predominant groups.

The frequency of carbapenem resistance genes in carbapenem-resistant and/or ESBL-*E. coli* isolates are presented in Table 3. In brief, a wide variety of carbapenem resistance *bla* genes were found to be circulating in pig populations in Asia and Europe. These included *bla*_*NDM–*1, NDM–5_, _*VIM–*1, KPC–2_, _and_ _*OXA–*48_ in several Asian countries (China, India, and South Korea) (Table 3). *bla*_*VIM–*1_ and *bla*_*OXA–*181_ were present in pig populations in Germany and Italy (as part of a pan-European study) (Table 3). Carbapenem resistance genes were found to be circulating in *E. coli* isolates collected between 2001 and 2011 in South Korea and Germany (Table 3), hinting that these genes have been present in pig populations globally since a long time.

**TABLE 3:** Distribution of carbapenem resistant genes in carbapenem-resistant or ESBL*-E. coli* isolates in pigs based on literature review.

Country	Selection criteria	Year of collection	Number of isolates tested	Genes (number of isolates carrying the gene)	References
China	Carbapenem-R	2009–2014	22	*bla*_*KPC–*2_ (2), *bla*_*NDM*_ (0), *bla*_*IMP*_ (0), *bla*_*VIM*_ (0), *bla*_*OXA–*48_ (0)	Cheng et al., 2019
	Carbapenem-R	2015–2017	9	*bla*_*NDM–*5_ (9)	Ho et al., 2018
	Carbapenem-R	2014–2015	5	*bla*_*NDM–*1_ (5)	Lin et al., 2017
	ESBL positive	2015–2017	44	*bla*_*OXA–*48_ (4), *bla*_*KPC–*2_ (2), *bla*_*NDM–*1_ (1)	Liu et al., 2018
	ESBL positive	2006–2009	10	*bla*_*KPC–*2_ (0)	Tian et al., 2012
Europe	Carbapenem-R	2015–2016	11	*bla*_*OXA–*181_ (2), *bla*_*KPC*_ (0), *bla*_*NDM*_ (0), *bla*_*VIM*_ (0)	Pulss et al., 2017
Germany	ESBL positive	2011	221	*bla*_*VIM–*1_ (1)	Fischer et al., 2012; Roschanski et al., 2017
Greece	ESBL positive	Unknown	11	*bla*_*KPC*_ (0), *bla*_*NDM*_ (0), *bla*_*IMP*_ (0), *bla*_*VIM*_ (0), *bla*_*OXA–*48_ (0)	Athanasakopoulou et al., 2021
India	Carbapenem-R	2014–2016	23	*bla*_*NDM*_ (8), *bla*_*KPC*_ (0), *bla*_*IMP*_ (0), *bla*_*VIM*_ (0)	Pruthvishree et al., 2017
	Carbapenem-R	2016–2017	9	*bla*_*NDM–*1_ (1), *bla*_*NDM–*5_ (1)	Tamta et al., 2020
South Korea	ESBL positive	2001–2011	95	*bla*_*NDM–*1_ (14), *bla*_*KPC*_ (12), *bla*_*VIM*_ (5)	Han et al., 2016
	ESBL positive	2017–2020	161	*bla*_*NDM*_ (0), *bla*_*KPC*_ (0)	Lee et al., 2021

### Relative Abundance and Genomic Characteristics of Extended Spectrum Beta-Lactamase*/amp*C and Carbapenemase Genes

**FIGURE 7:**
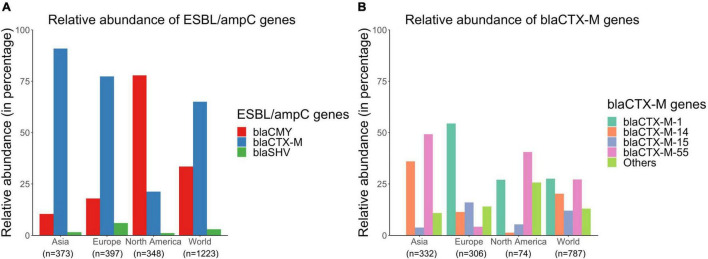
Relative abundances of **(A)** *bla*_*CTX–M*,_ *bla*_*CMY*_, and *bla*_*SHV*_ genes and **(B)** *bla*_*CTX–M*_ subtypes in publicly available *E. coli* genomes of swine origin.

Overall, there were 1,223 genomes out of the 6,167 sequences analyzed that carried ESBL/*amp*C genes (either *bla*_*CTX–M*_, *bla*_*CMY*_, or *bla*_*SHV*_ genes) globally. Details of these genomes are available in Supplementary File 4. *bla*_*CTX–M*_ genes were more prevalent compared to *bla*_*CMY*_ and *bla*_*SHV*_ genes in ESBL/*amp*C isolates from Asia (89.0% *bla*_*CTX–M*_, 10.5% *bla*_*CMY*_, and 1.61% *bla*_*SHV*_) and Europe (77.1% *bla*_*CTX–M*_, 17.9% *bla*_*CMY*_, and 6.04% *bla*_*SHV*_) (Figure 7A). In contrast, *bla*_*CMY*_ were the most prevalent genes in ESBL/*amp*C isolates in North America (77.9% *bla*_*CMY*_, 21.3% *bla*_*CTX*–*M*_, and 1.15% *bla*_*SHV*_) (Figure 7A).

Furthermore, there were several different subtypes of *bla*_*CTX–M*_ genes distributed globally in the sampled pig populations. *bla*_*CTX–M–*1_, *bla*_*CTX–M–*55_, and *bla*_*CTX–M–*14_ were the most prevalent *bla*_*CTX–M*_ genes present in 27.8, 27.5, and 20.8% of *bla*_*CTX–M*_ carrying isolates, respectively (Figure 7B). However, the distribution patterns of these *bla*_*CTX–M*_ subtypes varied across different continents (Figure 7B). For example, 40.5 and 50.3% of *bla*_*CTX–M*_-positive isolates carried *bla*_*CTX–M–*55_ genes in North America and Asia, respectively, whereas only 4.25% of *bla*_*CTX–M*_-positive isolates carried this gene in Europe (Figure 7B). Similarly, distributions of *bla*_*CTX–M–*1_, _–14_, _and –15_ genes also varied across continents (Figure 7B).

Only 22 of the *E. coli* genomes carried a carbapenemase gene. The most common type of carbapenemase gene was *bla*_*NDM–*5_, that was present in 13 Chinese isolates. Other carbapenemase genes were *bla*_*NDM–*1_ (China, *n* = 3), *bla*_*IMP–*27_ (Canada, United States, *n* = 3), *bla*_*IMP–*38_ (China, *n* = 1), *bla*_*VIM–*5_ (Belgium, *n* = 1) and *bla*_*GES–*5_ (Germany, *n* = 1). Since carbapenemase genes were so rare, we were not able to generalize their genetic characteristics and global distribution, but this information can be extracted from the Supplemental File 4.

### Characteristics of Isolates Carrying *bla*_*CMY*_ and *bla*_*CTX–M*_ Genes

Extended spectrum beta-lactamase/*amp*C isolates belonged to 319 unique sequence types (STs). Top five genetic characteristics of these isolates are presented in Table 4. Nearly 40% of the ESBL/*amp*C isolates belonged to clonal complex (CC) 10 (21.9%, including ST10 and ST744), CC23 (10.1%, including ST88 and ST410), and CC155 (7.3%, including ST58); and 75% of ESBL/*amp*C isolates were either phylotype A (43%) or B1 (32%).

**TABLE 4:** Top characteristics of publicly available *E. coli* genomes of swine origin carrying chromosomal mutations in ESBL/*amp*C genes.

Genotype	ST	Clonal complex	Phylotype	Serotype	*fim*H
ESBL/*amp*C (*n* = 1223)	10 (103), 58 (62), 100 (60), 101 (55), 88 (36)	10 (268), 23 (123), 155 (89), 165 (85), 101 (65)	A (526), B1 (391), C (116), G (49), D (42)	–:H10 (42), O8:H9 (37), O149:H10 (35), O9:H4 (30), –:H4 (29)	H54 (177), unknown (115), H23 (74), H32 (74), H24 (70)
*bla*_*CMY*_ (*n* = 409)	100 (46), 10 (37), 58 (32), 101 (19), 6449 (18)	10 (62), 165 (53), 23 (49), 155 (41), 101 (20)	A (159), B1 (123), C (46), G (17), D (17)	O149:H10 (27), –:H10 (24), –:H4 (21), –:H51 (18), O9:H4 (16)	Unknown (52), H54 (51), H23 (31), H32 (31), H24 (27)
*bla*_*CTX*–M_ (*n* = 787)	10 (63), 101 (32), 58 (30), 410 (27), 744 (27)	10 (201), 23 (69), 155 (48), 101 (41), 165 (33)	A (358), B1 (257), C (66), G (31), F (26)	O89:H9 (34), –:H9 (24), –:H12 (23), –:H23 (20), –:H10 (17)	H54 (117), unknown (63), H41 (45), H32 (43), H23 (42), H24 (42)

*Numbers in parentheses indicate the number of isolates.*

**FIGURE 8:**
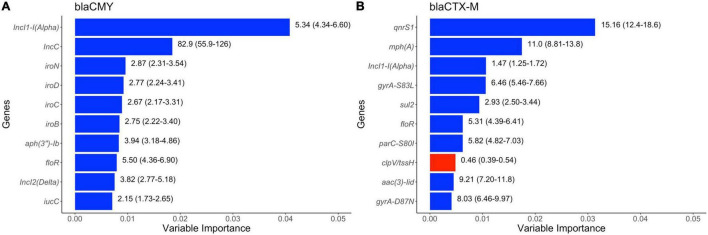
Top ten variables of importance predictive of **(A)** *bla*_*CMY*_ and of **(B)** *bla*_*CTX–M*_ genes in publicly available *E. coli* genomes of swine origin. Variable importance was estimated using Random Forest models. The numbers on the right side of the bars represent unadjusted odds ratio (95% confidence values). “Blue” and “red” colored bars represent positive (odds ratio > 1) and negative (odds ratio < 1) association between dependent and independent variables.

The results of random forest models are displayed in Figure 8. Specific plasmid replicon types (IncI1-alpha, IncC, and IncI2-delta), virulence factors (*iro* operon) and genes that can confer resistance to aminoglycosides [*aph(3′′)-Ib*] and florfenicol (*floR*) were the most common predictors of presence of *bla*_*CMY*_ gene in these *E. coli* genomes. Four genetic determinants of (fluoro)quinolone resistance (*qnrS1*, *gyrA*-S83L, *parC*-S80I, and *gyrA*-D87N) were among the top 10 predictors of *bla*_*CTX*–*M*_ genes in these genomes, with other AMR markers [*sul2*, *floR*, *aac (3)-II*, and *mph(A)*], plasmid replicon (IncI11-alpha) and virulence factor (*clpV/tssH*) being the other top 10 predictors of *bla*_*CTX–M*_ presence in isolates in swine populations.

## Discussion

The systematic literature review performed documented that the percent proportion estimates of phenotypic 3GC resistance in swine *E. coli* were variable depending on the countries and regions, as well as the source (healthy or diseased animals) from where an isolate was sampled. In general, the frequencies of 3GC resistance were low in isolates from Australia and European and North American countries. Some of these countries have imposed policies restricting the use of antimicrobials in animal production, including cephalosporins (Agerso and Aarestrup, 2013; Abraham et al., 2015; Callens et al., 2018), and this might have played a role in lower occurrence of 3GC resistance in generic *E. coli* isolates in pigs. Indeed, Chantziaras et al. (2014) found a high correlation between antimicrobial use and resistance using data available at a national level from seven European countries (Chantziaras et al., 2014). In contrast, the proportions of 3GC resistance were strikingly high in *E. coli* isolates recovered from swine in Asian countries. Asian countries have been known for higher rates of antimicrobial usage in animal production compared to other regions of the world (Van Boeckel et al., 2015). Most Asian countries however have yet to institute structured programs to monitor antimicrobial usage and surveillance of AMR (Founou et al., 2016). The results from our systematic review further emphasize the critical and immediate need to establish policy changes in Asian countries, especially regarding monitoring the use of critical antimicrobials such as 3GCs in food animals, specifically swine production. Comparing maps of sampling indices and pooled proportions of 3GC resistance, it is evident that the sampling efforts are inversely proportionate to the frequency of 3GC resistance and countries with lower number of isolates have higher proportions of 3GC resistance.

The results of this analysis also strongly suggest that a low proportion of 3GC resistance does not imply that the fecal carriage rate (i.e., prevalence at animal-level) is also low. Although fecal carriage rates are not as often discussed as isolate-level frequencies of AMR, these rates can provide valuable epidemiological and microbiological information pertinent to AMR surveillance. Fecal carriage rate is an additional important metric for measuring the extent of spread of AMR in animal populations. For example, in nationwide surveillance conducted by Swedish and Norwegian researchers between 2011 and 2019, the frequency of 3GC resistance seems to be stable and extremely rare but the fecal carriage rates are seemingly on a rise (from 10 and 5% in 2011 to 19 and 12% in 2019 from fecal samples collected in Norway and Sweden, respectively) (NORM/NORM-VET, 2012, 2020; SVARM, 2012; SWEDRES-SVARM., 2020). Secondly, screening of samples using selective screening can aid in early discovery of rarer but critical mechanisms of AMR and bacterial isolates carrying these genes. For example, in the same studies, Swedish and Norwegian researchers discovered that the increase in fecal carriage rates was due to mutations in promoter regions of chromosomal *amp*C genes rather than transferable ESBl/*amp*C genes. This discovery would not have been possible if the scientists had relied on the rather small number of 3GC resistant isolates collected during non-selective surveillance. It should be noted that different media and concentrations of antimicrobials were used for estimating fecal carriage rates in the studies we extracted data from and these differences can hinder the estimation of true prevalence of cephalosporin and carbapenem resistance (Duggett et al., 2020; Jacob et al., 2020).

Although the phenotypic occurrence of carbapenem resistance in swine populations was found to be very low, the significance of occasional studies reporting higher values should not be understated. The widespread dissemination of carbapenem resistance is a relatively recent phenomena as evidenced by the post-2010 changes in the extent and prevalence of carbapenem resistance in bacterial populations in European countries (Albiger et al., 2015). Considering the mechanistic similarities in propagation and dissemination of extended spectrum cephalosporin and carbapenem resistance (such as plasmid mediated carriage of genetic elements, broad range substrate specificity, broad bacterial host range, etc.), carbapenem resistance could become established in bacterial populations as has been the case with resistance against extended spectrum cephalosporin in *E. coli* from swine. Some studies on pigs already indicate that carbapenem resistance might be highly prevalent in certain countries such as Thailand and Ghana (Pokhrel et al., 2018; Dawangpa et al., 2021; Larbi et al., 2021). Also, fecal carriage rate of greater than 1% in pigs from China and India should be of significant concern. Thus, there is an active need for assessing the risk of food animals and food products, such as swine products, as a source of carbapenem resistance (Madec et al., 2017; Kock et al., 2018).

The global pattern of relative gene distribution of *bla*_*CTX–M*_ subclasses in pigs differed from that in humans. In humans, *bla*_*CTX–M–*14_ and *bla*_*CTX–M–*15_ are the most prevalent of *bla*_*CTX–M*_ genes, with *bla*_*CTX–M–*27_ identified as an emerging subclass (Bevan et al., 2017) which is in contrast to our results, where *bla*_*CTX–M–*1_ and *bla*_*CTX–M–*55_ were the most prevalent *bla*_*CTX–M*_ genes. This difference can be attributed to the evolutionary success of pathogenic ST131 isolates which also carry *bla*_*CTX–M–*14, –15_, _and__–27_ genes in humans (Nicolas-Chanoine et al., 2014; Matsumura et al., 2015), whereas in pigs, *E. coli* ST 131 isolates are rarely isolated (Reid et al., 2019; Flament-Simon et al., 2020; Hayer et al., 2020a), and *bla*_*CTX–M*_ genes were present on multiple ST types with the most common being ST10.

*bla*_*CMY*_ genes were the most common ESBL/*amp*C genes in North America. However, there has been a rise in *bla*_*CTX–M*_ genes, which had not been reported in swine populations since the beginning of 2010s (Roach and Wallinga, 2013). Future studies should focus on whether there is a further turnover of ESBL/*amp*C genes in North America and if there is any impact of these changes on swine and human health. Finally, the genomic dataset currently available might not be representative of the distribution of the *bla* genes that can confer carbapenem resistance. Several studies have reported the presence of *bla*_*VIM*–1, –IMP–1, –KPC, –NDM–5_, _and_ _–OXA–181_ in pig populations across the world (Tang et al., 2011; Fischer et al., 2012; Han et al., 2016; Pulss et al., 2017; Roschanski et al., 2017). Clearly, there is a paucity of genomic data when it comes to carbapenem resistance in pig populations. This is a major knowledge gap considering the importance of carbapenem resistance and the fact that it could only take a certain “right” combination of *E. coli* ST type, resistance gene(s) and plasmid to unleash the next highly resistant, virulent, pandemic *E. coli* clone, as has been the case with the hyper-virulent, fluoroquinolone and cephalosporin resistant ST131 clones (Stoesser et al., 2016).

The most noteworthy result from Random Forest models was a strong association of *bla*_*CTX–M*_ genes with both plasmid-mediated and chromosomal-mediated mechanisms of fluoroquinolone resistance. Co-resistance against multiple drug classes makes the problem of AMR in bacteria even more complex, as use of one class of antimicrobials can provide positive selection pressure to maintain resistance against other antimicrobial classes (Holmes et al., 2016). Fluoroquinolones are also considered as critically important antimicrobials in human medicine (World Health Organization., 2019) and are also used in food animals in some countries for treatment purposes (Römer et al., 2017; Ahmad et al., 2021). Co-resistance to fluoroquinolones and 3GCs can hence be considered both a human and animal health threat. Previous studies have noted an increased co-resistance between these two antimicrobials at a regional or national level (Balkhed et al., 2013; Bai et al., 2015; Falgenhauer et al., 2016; Pires et al., 2021) and our results confirm that this co-resistance is widespread across multiple countries and can be considered to be a global phenomenon.

This study aimed at providing a comprehensive review on the global frequency of distribution of 3GC and carbapenem resistance in swine *E. coli* populations. However, there are several limitations that should be kept in mind while interpreting the results from this analysis. First, limiting articles to English-only might have excluded data from certain countries, thus introducing a publication language bias. A high number of articles did not report if the pigs were administered antimicrobials for growth promotion, disease prevention, therapy, or control. Antimicrobial use is arguably the most-often cited risk factor in selection of AMR and it has been shown that isolates from farms that do not use antimicrobials are less likely to be resistant as compared to conventional farms (Osterberg et al., 2016). Similarly, husbandry factors such as level of intensification has been correlated with the extent of AMR in developing countries (Strom et al., 2018; Lunha et al., 2020). The vast majority of the studies did not report which sampling scheme was employed during isolate selection, making the direct comparison between countries with and without surveillance programs difficult. Indeed, results from countries without a surveillance program were more likely to be statistically heterogeneous than countries with an active surveillance system with a well-designed sampling scheme. Some of the studies included were point prevalence surveys and data from such surveys might not be representative of the situation in the country as a whole. Finally, we did not include temporality in the meta-analyses and we were not able to incorporate impact of country-level policy changes regarding antimicrobial usage in our models because of data scarcity from several nations. We were not able to assess the effect of time on estimates of pooled proportions of AMR because data at a country-disease level was too scarce to analyze this effect and pooling AMR estimates from different time-points could have contributed to statistical heterogeneity in some cases.

There are several limitations of the genomic meta-analysis as well. Several plasmid-level genetic features can be investigated further to confirm the results of the random forest models. For example, we identified an association between *bla*_*CTX–M*_ genes and *sul2, floR*, and *mph(A)*. These genes have been often associated with other genetic elements such as integrons and insertion sequences which aid in transmission and co-persistence of multiple AMR genes. *mph(A)* have been found to be associated with class I integrons in isolates carrying ESBL genes (Hastak et al., 2020; Cameron et al., 2021). Insertion sequences have and continue to play a massive role in mobilization and evolution of *bla* genes (Cantón and Coque, 2006). Constructing the genetic contexts of *bla* genes from the contigs of the bacterial genomes can help in identifying these mobile genetic elements and associated AMR genes and verifying the results of random forest models.

Despite the above limitations, we believe that we have achieved the aim of being comprehensive while acknowledging and correcting certain biases. We are also sharing the phenotypic and genomic data in Supplementary Files 2, 4 should other researchers be interested in evaluating the results with a different approach.

## Conclusion

This review summarizes the frequency and distribution of phenotypic and genotypic 3GC and carbapenem resistance and associated AMR genes in *E. coli* isolates collected from swine worldwide. We observed a large variability in proportions of *E. coli* resistant to 3GCs and distribution of associated gene markers in swine *E. coli*. Carbapenem resistance remains low but should be continuously monitored. We also discussed the strengths and weaknesses of the current review in order to give the full context of this complex issue to the readers. This review is useful in summarizing the continually evolving problem of AMR in swine production and reinforces the need to review current country and global policies.

## Author Contributions

SH and JA conceived the review. SH and AN selected the databases and search strings. SH, AC-H, and EP retrieved the manuscripts and collected the data. SH, AC-H, EP, AN, EE, JB, TJ, AP, and JA contributed in data analysis and writing and editing manuscript. All authors contributed to the article and approved the submitted version.

## Conflict of Interest

The authors declare that the research was conducted in the absence of any commercial or financial relationships that could be construed as a potential conflict of interest.

## Publisher’s Note

All claims expressed in this article are solely those of the authors and do not necessarily represent those of their affiliated organizations, or those of the publisher, the editors and the reviewers. Any product that may be evaluated in this article, or claim that may be made by its manufacturer, is not guaranteed or endorsed by the publisher.

## Supplementary Material

Supplementary File 1 Database search strings.

Supplementary File 2 Raw data collected from articles.

Supplementary File 3 Detailed pooled proportion estimates of phenotypic 3GC and ceftazidime resistance.

Supplementary File 4 Genotypic data including metadata from Enterobase, virulence genes, plasmid replicon types and resistance genes.
